# Adeno-Associated Virus Type 2 and Human Adenovirus Species F Type 41 Co-infection Associated with Acute Severe Hepatitis in Children, California, USA

**DOI:** 10.3201/eid3207.260284

**Published:** 2026-07

**Authors:** Ran Zhuo, Colette J. Matysiak Match, Sanchi Malhotra, Huan Vinh Dong, Kristina Adachi, Robert S. Venick, Grace Aldrovandi, Shangxin Yang

**Affiliations:** University of California Los Angeles David Geffen School of Medicine, Los Angeles, California, USA (R. Zhuo, C.J. Matysiak Match, S. Malhotra, H.V. Dong, K. Adachi, R.S. Venick, G. Aldrovandi, S. Yang); Duke University School of Medicine, Durham, North Carolina, USA (R. Zhuo); University of California San Francisco School of Medicine, San Francisco, California, USA (C.J.M. Match)

**Keywords:** viruses, adenovirus, adeno-associated virus type 2, human adenovirus F41, pediatric hepatitis of unknown etiology, liver failure, California, United States

## Abstract

Since late 2021, clusters of acute severe hepatitis of unknown etiology in previously healthy children, including some requiring liver transplantation, have been reported worldwide. Co-infection with adeno-associated virus type 2 (AAV2) and human adenovirus species F type 41 (HAdV-F41) has been identified in most cases. Global incidence peaked in 2022, and pediatric liver failure involving co-infection with AAV2 and HAdV-F41 has remained rare in recent years. We report 2 cases of pediatric liver failure associated with AAV2 and HAdV-F41 in California, USA, in March 2024 and January 2025. The patients had high adenovirus loads (393,000 and 480,000 copies/mL), extended adenovirus viremia (2 and 3.5 months), and high AAV2 viral loads (1.3 and 1.0 × 10^6^ copies/mL). One patient required liver transplantation; both patients recovered. Our findings underscore the need for heightened physician awareness and expanded surveillance to identify and characterize new cases, improve understanding of underlying pathophysiology, clarify risk factors, and inform therapeutic strategies.

Since late 2021, clusters of severe acute hepatitis in children have been linked to adeno-associated virus type 2 (AAV2) and co-infection with helper viruses, most frequently human adenovirus species F type 41 (HAdV-F41) but also Epstein–Barr virus (EBV), human herpesvirus 6, or both (*1*–*6*). This unexpected surge of severe disease, uncharacteristic of AAV2 infection alone, has been hypothesized to result from altered patterns of childhood viral exposure during the COVID-19 pandemic, possibly coupled with host immunogenetic susceptibility (*3*,*7*). More than 1,000 suspected cases of severe acute hepatitis across 35 countries were reported to the World Health Organization (WHO); ≈6% of patients required liver transplantation, and the mortality rate was 1.8% (*8*–*10*). Environmental surveillance (e.g., wastewater sampling) has demonstrated parallel peaks in community circulation of AAV2 and HAdV-F41 during outbreak periods (*11*,*12*). By June 2023, the outbreak seemingly dissipated, with only rare reports of AAV2-associated hepatitis (*13*). We report 2 cases of acute non-A–E hepatitis with liver failure in previously healthy 3-year-old children in California, USA, in March 2024 and January 2025.

## The Cases

### Case 1

In March 2024, a previously healthy girl, 3 years and 8 months of age, was brought to a hospital 3 days after she had jaundice scleral icterus, nonbloody nonbilious emesis, and fatigue develop. Over the next 2 days, pale stools and dark urine had developed. At home, her 2 older siblings reportedly had conjunctivitis, but she remained afebrile without sore throat, cough, congestion, abdominal pain, diarrhea, rash, or conjunctivitis. She had not recently taken new medications, specifically no acetaminophen, and no suggestion of accidental substance ingestion was apparent. Her birth, medical, surgical, and family histories were unremarkable for liver, autoimmune, or metabolic disease, as well as for immunodeficiency or recurrent infections. She had no allergies, and her immunizations were up to date except for COVID-19. Social and exposure history included household exposure to dogs and cats.

On examination, she was comfortable and afebrile (36.3°C). Heart rate was 110 beats/min, respiratory rate 27 breaths/min, blood pressure 98/59 mm Hg, and oxygen saturation 95% on room air. Findings included scleral icterus and jaundiced skin; results of cardiopulmonary and neurologic examinations were unremarkable. Laboratory results showed leukocyte count of 7.46 × 10^9^ cells/L (reference range 5.0–16.0 × 10^9^ cells/L), hemoglobin 12.4 g/dL (reference range 11.5–14.0 g/dL), platelets 90 × 10^9^/L (reference range 140–400 × 10^9^/L); alanine transaminase (ALT) was 3,667 U/L (reference range 8–24 U/L), aspartate transaminase (AST) 4,945 U/L (reference range 20–39 U/L), total bilirubin 10.8 mg/dL (reference range 0.2–0.8 mg/dL), direct bilirubin 9.2 mg/dL (reference range <0.2 mg/dL), prothrombin time (PT) 21 seconds (reference range 12.1–14.5 seconds), partial thromboplastin time (PTT) 32.2 seconds (reference range 25.1–36.5 seconds), international normalized ratio (INR) 1.9 (therapeutic range 2–3), ferritin 3,612 ng/mL (reference range 8–180 ng/mL), ammonia 35 µmol/L (reference range 18–90 mcg/dL), and gamma glutamyl transferase (GGT) 108 U/L (reference range 7–68 U/L).

Results of standard viral hepatitis panels (A–E), herpesvirus testing, and BioFire FilmArray Gastrointestinal and Respiratory panels (bioMérieux, https://www.biomerieux.com) including HAdV as a target were all negative; QuantiFERON-TB Gold (QIAGEN, https://www.qiagen.com) result was indeterminate (Table 1). Abdominal ultrasound showed increased hepatic echogenicity without biliary obstruction.

**Table 1:** Initial infectious workups for 2 pediatric cases of acute severe hepatitis of unknown etiology associated with adeno-associated virus type 2 and human adenovirus species F type 41 co-infection, California, USA

Pathogen	Sample type	Testing modality	Result	
			Case 1	Case 2
Cytomegalovirus	Blood	PCR	Negative	Positive <35 copies/mL
Cytomegalovirus IgG and IgM	Blood	Serology	Negative	IgG positive, IgM negative
Epstein-Barr virus	Blood	PCR	Negative	Negative
	Blood	Monospot	Negative	Negative
Hepatitis A virus IgM	Blood	Serology	Negative	Negative
Hepatitis B virus surface antigen	Blood	Serology	Negative	Negative
Hepatitis B virus core antibody	Blood	Serology	Negative	Negative
Hepatitis C virus antibody	Blood	Serology	Negative	Negative
Hepatitis C virus	Blood	PCR	Negative	Not performed
Hepatitis E virus	Blood	PCR	Negative	Not performed
Hepatitis E virus IgG and IgM	Blood	Serology	Negative	Negative
Parvovirus B19	Blood	PCR	Negative	Negative
Enterovirus	Blood	PCR	Negative	Negative
Human herpes virus 6	Blood	PCR	Negative	Negative
Parechovirus	Blood	PCR	Negative	Negative
Varicella-zoster virus IgM	Blood	Serology	Negative	Negative
West Nile virus	Blood	PCR	Negative	Not performed
West Nile virus IgG and IgM	Blood	Serology	Negative	Not performed
Herpes simplex virus type 1 and 2	Blood	PCR	Negative	Negative
SARS-CoV-2	Mid-turbinate swab	PCR	Negative	Negative
*Bartonella henselae* IgG and IgM	Blood	Serology	Negative	Not performed
*Coccidioides* IgG/IgM EIA	Blood	Serology	Negative	Not performed
Cryptococcal antigen	Blood	Serology	Negative	Not performed
QuantiFERON-TB Gold*			Indeterminant	Indeterminant
Leptospira				Negative
BioFire Gastrointestinal Panel†	Stool	PCR	Negative	Positive, sapovirus
ePlex Respiratory Pathogen Panel‡	Nasopharyngeal swab	PCR	Negative	Positive, rhinovirus/enterovirus

*QuantiFERON-TB Gold, QIAGEN, https://www.qiagen.com. †BioFire FilmArray Gastrointestinal Panel, bioMérieux, https://www.biomerieux.com. ‡ePlex Respiratory Pathogen Panel, Roche, https://www.roche.com.

The patient was empirically treated with N-acetylcysteine, cefepime, and metronidazole. Autoimmune serologies revealed a positive antinuclear antibody titer (1:160) and elevated IgG for her age (1,360 mg/dL, reference range 320–990 mg/dL). Results were negative for liver kidney microsomal 1, liver cytosol type 1, mitochondrial, and smooth muscle antibodies.

On hospitalization day 2, her coagulation profile worsened; PT and INR rose in subsequent days to a peak of 51.9 seconds for PT and 5.6 for INR on day 4. A percutaneous liver biopsy was performed after her coagulopathy was corrected with fresh frozen plasma and recombinant coagulation factor 7a that demonstrated panlobular severe acute hepatitis with lymphocytic predominance and 70%–80% hepatocyte loss. No viral inclusions were seen, and an adenovirus antibody probe was negative.

On day 3, qualitative plasma PCR for adenovirus returned a positive result, prompting initiation of cidofovir (5 mg/kg/wk intravenously). Quantitative whole-blood adenovirus PCR (ARUP Laboratories, https://www.aruplab.com) on day 8 measured 393,000 copies/mL (log 5.6).

Given clinical and biochemical concern for progressive liver failure, she was listed for transplant on day 4 (with a calculated Pediatric End-Stage Liver Disease Score of 25). On day 5, hyperammonemia, altered mental status, and worsening coagulopathy developed, necessitating intubation and continuous renal replacement therapy for ammonia clearance; her transplant status was upgraded to 1A. She underwent ABO compatible, whole-graft orthotopic liver transplantation on day 10. Weekly cidofovir was continued before and after transplantation for 40 days because of persistent adenovirus viremia, although PCR of explanted liver tissue was negative for adenovirus.

### Case 2

In January 2025, a previously healthy boy, 3 years and 1 month of age, was brought to a hospital with a 1-day history of jaundice and scleral icterus. He had experienced 1 week of postprandial abdominal discomfort and 2 days of pale, foul-smelling stools. Three weeks before care was sought, he had 1 day of emesis and a subjective fever, without concurrent or subsequent respiratory symptoms, rash, conjunctivitis, or diarrhea. His medical history was only notable for mild speech delay of unknown etiology without concerns for other growth or developmental delay. There was no reported family history of liver, autoimmune, metabolic, disease, immunodeficiency, or history of recurrent infections.

At admission, he was found to be afebrile; vital signs included heart rate 115 beats/min, respiratory rate 22 breaths/min, blood pressure 98/56 mm Hg, and oxygen saturation 100% on room air. He appeared comfortable. Examination showed diffuse jaundice, scleral icterus, and hepatomegaly. Initial laboratory results included leukocytes 10.55 × 10^9^ cells/L, hemoglobin 10.6 g/dL, and platelets 281 × 10^9^/L. Other notable laboratory results were ALT 3,371 U/L, AST 5,651 U/L, total bilirubin 6.6 mg/dL, direct bilirubin 6.0 mg/dL, PT 17 seconds, PTT 33.4 seconds, INR 1.5, ferritin 694, GGT 82 U/L, and ammonia 44 µmol/L. Results of autoimmune hepatitis workup (smooth muscle, liver kidney microsomal 1, mitochondrial, systemic panel) and urine toxicology were negative. Routine infectious hepatitis panels were likewise negative (Table 1), except for plasma adenovirus, which was PCR positive at 49 copies/mL (from referring hospital); rhinovirus/enterovirus detected by a respiratory pathogen multiplex PCR; and sapovirus detected by BioFire FilmArray Gastrointestinal Panel. However, results of both initial respiratory and stool panels were negative for adenovirus. Cytomegalovirus was detectable by blood PCR but below the limit of quantification (<35 copies/mL).

The patient remained afebrile and clinically stable throughout his admission. Initial adenovirus testing suggested low risk for severe or disseminated disease, given the low plasma viral load (49 copies/mL) and negative respiratory and stool panel results. On hospitalization day 6, a percutaneous liver biopsy revealed marked lobular inflammation, frequent acidophil bodies, ballooned hepatocytes, and focal bridging necroinflammatory activity; bile ducts were spared. Mixed inflammatory infiltrates (predominantly lymphocytes with eosinophils, neutrophils, and plasma cells) were noted. Results of special stains (Trichrome, periodic acid–Schiff, α_1_-antitrypsin, iron) and immunohistochemistry for cytomegalovirus, adenovirus, and EBV (EBER) were all negative. Concern for acute liver failure prompted treatment with cidofovir (1 mg/kg on hospital days 7, 9, and 11) alongside standard hydration and probenecid for renal protection. Intravenous methylprednisolone (1 mg/kg 2×/d) was also initiated on day 7; the dose was lowered on day 11 (0.5 mg/kg 2×/d) and then transitioned to oral prednisolone on day 14. The patient tolerated cidofovir well without renal toxicities.

Subsequent testing demonstrated new adenovirus positivity in stool, and on day 11, repeat quantitative whole-blood adenovirus PCR showed 480,000 copies/mL. Despite persistent high-level viremia, liver enzymes steadily improved. To enable transfer to outpatient therapy, cidofovir was adjusted to 5 mg/kg/wk beginning on day 17. At discharge on day 18, jaundice and scleral icterus had markedly resolved, and transaminase levels had declined (AST 218 U/L; ALT 360 U/L), although quantitative adenovirus PCR returned 564,000 copies/mL after discharge. The patient was discharged on oral steroids with plans for weekly cidofovir infusions and weekly adenovirus PCR monitoring. Despite clinical improvement, adenovirus viremia persisted for 3 months; cidofovir was continued for 6 months given prolonged steroid wean (Figure).

**Figure:**
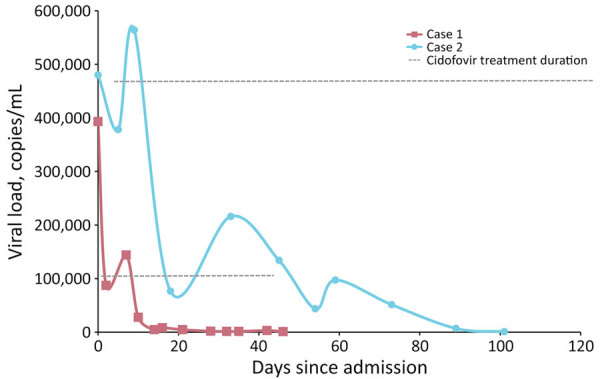
Longitudinal human adenovirus species F type 41 viral loads measured by quantitative PCR in whole-blood specimens from 2 pediatric cases of acute severe hepatitis of unknown etiology associated with adeno-associated virus type 2 and human adenovirus species F type 41 co-infection, California, USA. Viral loads are plotted against hospital day, illustrating high viremia preceding antiviral therapy and liver transplantation in case 1, the subsequent decline during cidofovir treatment, and persistent low-level detection despite clinical improvement in case 2.

## Materials and Methods

Our goal was to track the microbial etiology of acute hepatitis in both patients. We employed shotgun metagenomic next-generation sequencing (mNGS), targeted next-generation sequencing (tNGS), and quantitative PCR (qPCR) to track AAV2 load.

### Genomic DNA Extraction and Sequencing

We extracted DNA from whole blood, plasma, and liver tissue (liver tissue available only from patient 1) by using either the EZ1 Tissue Kit (QIAGEN) or the easyMAG system (bioMérieux). We then prepared indexed libraries on a MagicPrep NGS System (Tecan, https://www.tecan.com) and sequenced on a MiSeq platform (Illumina, https://www.illumina.com) using a 2 × 250 bp pair-end protocol. We analyzed raw reads with the Chan Zuckerberg ID (CZ ID) cloud-based metagenomic pipeline (https://www.czid.org).

### AAV2 Detection and Quantification by qPCR

We detected AAV2 by using a published primer-probe set (*14*). In brief, we performed real-time PCR in a 5-µL reaction volume containing 2.5 µL of 2X PrimeTime Gene Expression Master Mix (Integrated DNA Technologies, https://www.idtdna.com), 0.25 µL of AAV2-specific assay mix (10 µmol primers, 5 µmol probe), 1.25 µL of nuclease-free water (Thermo Fisher Scientific, https://www.thermofisher.com), and 1 µL of template DNA. We conducted amplification on an Applied Biosystems 7500 Fast Dx Real-Time PCR System (Thermo Fisher Scientific) under the following conditions: 95°C for 3 minutes, 40 cycles of 95°C for 5 seconds, and 60°C for 30 seconds. We quantified AAV2 viral load by using a standard curve generated from 10-fold serial dilutions of a custom oligonucleotide (Integrated DNA Technologies) with the following sequence: 5′-CGGCCTCAGTGAGCGAGCGAGCGCGCAGAGAGGGAGTGGCCAACTCCATCACTAGGGGTTCC-3′.

### Adenovirus tNGS and Genotyping

We performed adenovirus genotyping by targeted amplification and sequencing of the hexon hypervariable region using published primers (*15*). We performed PCR in a 20-µL reaction containing lyophilized High Fidelity PCR ecoDry Premix (TaKaRa Bio USA, https://www.takarabio.com), 1.6 µL of each primer (AD1 and AD2, 10 µmol), 16.8 µL of nuclease-free water (Thermo Fisher Scientific), and 5 µL of template DNA. We performed amplification on a ProFlex PCR System (Thermo Fisher Scientific) under the following conditions: 95°C for 1 minute; 35 cycles of 95°C for 30 seconds, 51°C for 1 minute, and 68°C for 1 minute; and a final extension at 68°C for 3 minutes.

We purified amplicons with AMPure XP beads (Beckman Coulter, https://www.beckman.com), according to the manufacturer’s instructions, and sequenced on an Illumina MiSeq platform, as we described. We processed sequence reads using the CZ ID pipeline and mapped resulting hexon sequences against the human adenovirus 41 reference genome (GenBank accession no. OR628209) using Geneious Prime 2023.2.1 (https://www.geneious.com). We confirmed genotype assignment BLAST analysis (https://blast.ncbi.nlm.nih.gov) against the National Center for Biotechnology Information nucleotide database. We deposited sequence files generated in this study into GenBank (BioProject no. PRJNA1455204).

## Results

For patient 1, shotgun mNGS identified AAV2 in both plasma and liver tissue. BLAST analysis revealed closest homology to AAV2 isolate CHC3511 (GenBank accession no. MK163942.1), and genome mapping analysis showed 81.3% whole-genome coverage and 95.4% identity (Table 2). AAV2 qPCR demonstrated high viral loads (1.3 × 10^6^ copies/mL) in plasma and liver before liver transplantation and AAV2 clearance from plasma after transplantation. tNGS detected HAdV-F41 in whole blood but not in the liver, consistent with the PCR results. The HAdV-F41 strain bears the closest similarity to human adenovirus 41 isolate 2330/N.Nov/RU/2009 hexon gene, partial coding sequence 2330/N.Nov/RU/2009 (GenBank accession no. HM588740.1; 100% coverage, 96.7% identity). Adenovirus quantitative real-time PCR at a reference laboratory (ARUP Laboratories) demonstrated high-level HAdV-F41 viremia before transplantation and a decline during antiviral therapy but persistent low-level detection for weeks thereafter (Figure). The patient experienced an episode of mild T-cell mediated rejection 1 month after transplantation, treated with intravenous steroid pulse for 1 week. She was discharged on day 36 receiving tacrolimus, prednisolone, and antimicrobial prophylaxis, with plans to continue weekly cidofovir until blood adenovirus PCR was negative (Figure). She was weaned off steroids and managed on tacrolimus monotherapy at 12 months after transplantation. At 2 years after transplantation, she had maintained normal biochemical graft function with no recent hospital admissions.

**Table 2:** Genomic analysis for 2 pediatric cases of acute severe hepatitis of unknown etiology associated with AAV2 and HAdV-F41 co-infection, California, USA*

Case no.	Source	NGS method	Virus identified	% Genome/ gene coverage	% Identity	Reference genome/gene	GenBank accession no.	
							Reference isolate	Patient isolate
1	Plasma and liver	Shotgun	AAV2	81.3	95.4	AAV isolate CHC3511_AAV.FL.circular, complete genome	MK163942	SRX33017783
	Whole blood	Amplicon (hexon gene)	HAdV-F41	100	96.7	HAdV-F41 isolate 2330/N.Nov/RU/2009 hexon gene, partial cds	HM588740	SRX33034982
2	Plasma	Shotgun	AAV2	35.1	90.9	AAV2, complete genome	NC_001401	SRX33017784
	Whole blood	Amplicon (hexon gene)	HAdV-F41	86	99.5	HAdV-F41isolate 2330/N.Nov/RU/2009 hexon gene, partial cds	HM588740	SRX33034983

*AAV2, adeno-associated virus type 2; HAdV-F41, human adenovirus species F type 41; cds, coding sequence; NGS, next-generation sequencing.

For patient 2, we identified AAV2 in plasma by using shotgun mNGS. BLAST analysis revealed closest homology to the AAV2 complete genome (GenBank accession no. NC_001401); genome mapping analysis showed 35.1% whole-genome coverage and 90.9% identity (Table 2). We also identified AAV2 in plasma (1 × 10^5^ copies/mL), stool (1 × 10^5^ copies/mL), and whole blood (1 × 10^6^ copies/mL) by qPCR. Targeted sequencing of the adenovirus hexon hypervariable region identified HAdV-F41 in whole blood with the closest homology to HAdV-F41 isolate 2330/N.Nov/RU/2009 hexon gene, partial coding sequence (accession no. HM588740; 86% query coverage and 99.5% identity).

Clinically, the patient’s hepatic function panel normalized biochemically by late February 2025. Prednisone and cidofovir were continued for 6 months at gradually lower doses (for prednisone) and longer intervals between infusions (for cidofovir). Treatments were discontinued in July 2025, and the patient continued to have normal liver function tests for the next 6 months.

## Discussion

An outbreak of acute severe hepatitis of unknown etiology (ASHUE) predominantly in children <5 years of age was first recognized in 2021 (*8*,*16*). AAV2, a dependoparvovirus that requires a helper virus (most commonly adenoviruses or herpesviruses) for productive replication, has been implicated (*17*,*18*). In the ASHUE cases, HAdV-F41 commonly provides this helper function. Proposed pathogenic mechanisms include a tropism shift of HAdV-F41 permitting hepatic co-infection and activation of AAV2 replication in hepatocytes and immune-mediated injury, potentially driven by high AAV2 antigen loads in the liver and shaped by host human leukocyte antigen (HLA) genotype (e.g., enrichment for HLA-DRB1*04:01) (*1*,*3*,*19*). Initial surveillance in the United Kingdom detected adenovirus in 68% of ASHUE cases, predominately type 41F (*20*). An early case series from the United States found adenovirus in 8 (89%) of 9 children with ASHUE and detected 3 distinct HAdV-F41 variants among 5 sequenced isolates; none showed viral inclusions or positive immunohistochemical staining in liver biopsies (*21*). In a larger analysis of >200 pediatric patients with hepatitis of unknown etiology over a 7.5-month period, only 45% of cases were positive for adenovirus (*16*), so adenovirus infection alone did not explain that outbreak. That analysis also investigated whether the hepatitis resulted from change in immune response secondary to COVID-19 or COVID-19 vaccination, but <40% of patients had evidence of ever having been infected with SARS-CoV-2, and only 4% had received >1 vaccine dose (*16*).

In the 2 cases we report, liver biopsies were negative for adenovirus by immunohistochemistry. Moreover, initial microbiological results identified high levels (10^5^–10^6^ copies/mL) of adenovirus in patient blood but failed to detect adenovirus in the explanted liver tissue from patient 1, supporting the likelihood that HAdV-F41 did not directly cause severe hepatitis in the children. Of note, patient 1 experienced a very rapid disease course, just 3 days from symptom onset to fulminant liver failure, whereas prior AAV2 case series reported prodromal period lasting 2–12 weeks (*3*). The 2 patients also had significantly higher adenovirus levels (393,000 copies/mL and 480,000 copies/mL) than were found in previous reports, which showed a maximum of 156,400 copies/mL (*21*). Patient 1’s duration of adenovirus viremia for 2 months was likely affected by need for increased immunosuppression after liver transplantation. Patient 2’s profound adenovirus viremia persisted for 3.5 months despite rapid clinical recovery and improved transaminase levels. Prolonged corticosteroid usage is suspected to have contributed to the prolonged viremia, although duration was longer than typically observed on antiviral treatment.

HAdV-F41 is an enteric adenovirus classically associated with acute gastroenteritis. Early in both cases we report, stool specimens were negative for adenovirus, but patient 2 later shed adenovirus in stool on hospital day 11, despite earlier detection of high-level viremia (10^5^–10^6^ copies/mL) in whole blood. Delayed enteric detection alongside marked viremia suggests possible altered tropism for HAdV-F41. Given that AAV2 integrates into human chromosome 19 and human peripheral blood mononuclear cells and hepatocytes serve as targets (*22*–*25*), we hypothesize that HAdV-F41 acts as a helper in hematopoietic cells, amplifying AAV2 replication that subsequently infects hepatocytes either directly causing liver injury or indirectly via immune-mediated mechanisms (*26*). That possibility is supported by the findings of high AAV2 loads (≈10^6^ copies/mL) in liver tissue despite undetectable HAdV-F41 in the same sample. After transplantation, AAV2 cleared rapidly in plasma in parallel with clinical recovery, whereas adenovirus viremia persisted for weeks, further implicating AAV2 as the primary driver of liver injury. After discharge, we did not obtain sufficient specimens from patient 2 for AAV2 quantification in blood. Future in vitro studies of the HAdV-F41 and AAV2 strains isolated from the patients are warranted to elucidate their cell tropism, cellular pathogenesis, and underlying mechanisms contributing to ASHUE in children. 

Multiple case–control studies employing mNGS have implicated AAV2 in acute pediatric hepatitis (*1*–*3*). In 1 US cohort, 93% of affected children tested positive for AAV2, compared with only 3.5% of matched controls (*1*). Another study found AAV2 in all 5 patients who required liver transplantation (*2*). Historically, AAV2 has been viewed as hepatotropic, and transient hepatitis after AAV-based gene therapy vectors is well documented (*27*). Given AAV2’s known liver tropism, those observations strengthen the hypothesis that, under specific conditions, primary AAV2 infection can precipitate severe liver injury. Seroprevalence of AAV2 peaks among children 3–5 years of age (*3*), aligning with both our cases and the median age in the 2021–2022 outbreak and suggesting that AAV2-associated hepatitis reflects acute rather than reactivated infection.

One study identified class II HLA-DRB1*04:01 variant allele as a risk factor for AAV2-associated hepatitis (*3*). In the cases we report, patient 1 was found to have the HLA-DRB1*04:07 variant allele, differing by only 2 amino acids by preliminary typing. However, high-resolution NGS is needed for confirmation. Further investigation is required to delineate the molecular mechanisms by which specific HLA class II variants confer increased susceptibility and to define the spectrum of immunogenetic risk alleles in AAV2-related liver disease.

Clinically, given the relatively high severity of illness among children with ASHUE associated with AAV2–HAdV-F41, many patients are treated with cidofovir and concomitant intravenous steroids. Steroids are used to control dysregulated immune responses; for some patients, the treatment normalizes liver function tests completely, and they can be weaned off steroids altogether. Other patients, however, remain immune-reactive and could go on to develop autoimmune hepatitis, requiring long-term maintenance immunosuppression and vigilant follow-up.

In conclusion, we identified HAdV-F41 and AAV2 co-infections in 2 pediatric ASHUE cases in California in 2024 and 2025, years after global outbreaks subsided in 2022. AAV2 testing is not readily available in most clinical settings, but our case studies suggest it remains in circulation and should be considered as a co-pathogen in ASHUE cases. The cases we report highlight the importance of equipping clinical laboratories with molecular tools such as PCR and NGS to identify HAdV-F41 and AAV2 infections in children who have non-A–E hepatitis. Beyond laboratory diagnosis, clinicians are encouraged to report unexpected ASHUE cases or clusters to public health authorities, even in the absence of official reporting requirements, because that practice is critical for effective surveillance. Our findings underscore the need for heightened physician awareness for pediatric liver failure associated with AAV2 and HAdV-F41 and expanded surveillance to promptly identify and characterize new cases, improve understanding of the underlying pathophysiology, clarify risk factors, and inform future therapeutic strategies.
